# Patients with COVID‐19 share their experiences of recovering at home following hospital care transitions and discharge preparation

**DOI:** 10.1111/hex.13595

**Published:** 2022-09-22

**Authors:** Joanne Ganton, Amberley Hubbard, Katharina Kovacs Burns

**Affiliations:** ^1^ Alberta Health Services Primary Health Care Edmonton Alberta Canada; ^2^ Alberta Health Services Clinical Quality Metrics/Data & Analytics Edmonton Alberta Canada; ^3^ School of Public Health University of Alberta Edmonton Alberta Canada

**Keywords:** COVID‐19, discharge, home to hospital to home, hospital to home transitions, patient experience, patient resources, recovery at home

## Abstract

**Introduction:**

Patients discharged following hospitalization for COVID‐19 require clear discharge protocols, information resources and communications to adequately prepare them to safely and successfully transition from hospital to home. Our study focuses on the patients' transition to recovering at home including their hospital discharge preparation and hospital experiences.

**Methods:**

A qualitative descriptive study design involved interviewing patients who had been hospitalized for COVID‐19 in one urban Alberta, Canada centre. Purposive sampling was used to select patients from a centralized COVID‐19 hospital patient database stratified by month between March 2020 and February 2021. Other inclusion criteria (e.g., sex and age) were also considered. Semi‐structured interviews with patients were recorded, transcribed and analysed using thematic analysis. Data sufficiency and saturation were determined.

**Results:**

Twelve patients shared their lived experiences and recovery journey from COVID‐19. Themes were reported under three main areas as framed by the study aim—the current status of patients recovering at home, including the supports they used to manage; their discharge process and preparation to go home; and their various hospital‐related experiences. Suggestions for improving aspects of the patient journey were also captured.

**Conclusion:**

Findings provided details of the needs, information gaps and what matters most to patients when they are recovering from COVID‐19 at home, including their preparation to safely and successfully transition from hospital to home (i.e., feeling well prepared to go home, including being adequately assessed and having clear discharge protocols and communication). Key learnings were applied to improve or develop patient discharge and transition resources.

**Patient or Public Contribution:**

A patient/family advisor and patient experience partners were involved throughout the study, codeveloping all aspects, from the study design to the reporting and application of the findings. Leading into the study, patient experiences and feedback regarding the home from hospital recovery journey informed multiple aspects, including the codevelopment of the interview guide.

## 1 INTRODUCTION

During the COVID‐19 pandemic, concerns for those individuals diagnosed and hospitalized with COVID‐19 included not only clinical and physical but also mental health (i.e., psychological, emotional and social well‐being) experiences associated with having COVID‐19 and related public restrictions.^1^, ^2^, ^3^, ^4^ These latter experiences included fear of the unknown, loneliness related to isolation or being quarantined with no in‐person family contact, mental distress and anxiety related to having COVID‐19 and fighting to survive it, and uncertainty related to outcomes.^5^, ^6^

Studies that focused on patients' discharge from the hospital generally found their experiences to be less positive than their hospital experiences with family or friend visitation restrictions and less availability of physiotherapy or other supports.^7^, ^8^, ^9^ Some patients felt that their discharge was rushed and they were not well prepared to go home, including being inadequately assessed to be discharged.^10^, ^11^ Many had not received any information about the discharge arrangements with family or caregivers, including arranging for transportation home, having contacts for further support or advice as needed, or what health advice or further treatments to follow‐up while recovering at home.^10^, ^11^ Having clear discharge protocols and communication with patients and their families/caregivers was viewed as critical,^12^ particularly if there needed to be regular monitoring of patients to assess their clinical outcomes or the need for medical or rehabilitation care,^12^, ^13^, ^14^, ^15^ oxygen support or virtual or other types of care.^16^, ^17^, ^18^ The intent of such monitoring during recovery was to address patient needs and reduce readmission to the hospital.^19^, ^20^ For some patients, there was increased clinical deterioration after being discharged, and a high chance of being readmitted to the hospital within 60 days.^21^, ^22^

Very few studies followed COVID‐19 patients through their experience journey, starting with where they were at with recovering at home and reflecting back on their experiences with their hospital discharge and care. This reversed approach to the conversation was intentional to demonstrate to patients our empathy regarding their recovery from COVID‐19. The approach also provided patients some time to establish trust and become comfortable talking about their recovery, and remembering their experiences, some very emotional, as they reflected back on their hospital discharge and entire care journey. We wanted to explore specific experiences that influenced their recovery, including gaps in information and person‐centred care to address their needs at the time of discharge or while hospitalized. The aim of our study was to understand these lived experiences of patients with COVID‐19 as they continue their recovery at home following their transition from the hospital. Through these experiences, we intended to inform improvement initiatives related to various patient supports, information, resources and practices, which would more appropriately guide and prepare patients for their discharge from the hospital, and their recovery at home, including follow‐up care and/or self‐management.

## 2 METHODS

### 2.1 Study setting

The study setting was Alberta Health Services (AHS), the largest provincial health system in Canada. More specifically, our study was focused on the Calgary Zone, one of five zones within AHS that had designated COVID‐19 treatment hospitals throughout the pandemic. Table 1 provides some statistics for the Calgary Zone regarding patients hospitalized for COVID‐19 in total, and by month (between 1 March 2020 and 1 February 2021), as well as by age and gender. Leadership within the Calgary Zone identified a need to explore the experiences of patients hospitalized with COVID‐19. A telephone survey was initially conducted with 329 patients hospitalized with COVID‐19 between March 2020 and January 2021. These survey results (not reported here), along with the literature helped inform the aim and design of this study.

**Table 1:** Setting context data

Inclusion criteria	Total number of patients hospitalized and discharged (*n* = 1645 of which 352 were in ICU)
Month	
March 2020	47
April	103
May	59
June	28
July	27
August	28
September	54
October	70
November	348
December	493
January 2021	388
Total	1645
Sex/gender	
Male	917 (56%)
Female	728 (44%)
Other	
Age	
18–44	355 (22%)
45–64	500 (30%)
65–79	436 (27%)
80+	354 (22%)
Average length of stay	
Non ICU units	Range is 3.9 days in March 2020 to 19.6 days in June 2020
ICU	Average 11.7 days Average 12.8 days

*Note*: Total number of patients hospitalized and discharged, and number of patients by month, age and sex/gender in Calgary Zone.

Abbreviation: ICU, intensive care unit.

### 2.2 Study design/methods

A qualitative descriptive design^23^ was chosen to explore the in‐depth experiences of patients hospitalized with COVID‐19 between March 2020 when AHS first officially diagnosed COVID‐19 patients, and February 2021 when the third wave of COVID‐19 was starting to taper off.

### 2.3 Participants

As per qualitative studies involving the gathering of lived experiences of individuals regarding a specific situation or circumstance, it was difficult to predict or pre‐determine how many patients would need to be interviewed regarding their lived experiences recovering from COVID‐19 following their hospitalization and discharge home. We used purposeful sampling^24^ of English‐speaking patients hospitalized and discharged from Calgary hospitals between 8 March 2020 and 1 February 2021. Although our main intent was to have participants selected for each of the months in our targeted timeframe, our selection of participants was also guided, but not stratified by other inclusion criteria as shown in Table 2 (e.g., sex, age, where a person lives). We initially anticipated completing 11 interviews, but the final number of interviews was determined by data saturation through the iterative thematic analysis of transcribed data described under Section 2.5.

**Table 2:** COVID‐19 hospitalized patient interview inclusion criteria

Criteria	Details
Month during patient hospitalization—One in each month if possible and more in months during increased patient hospitalizations.	March 2020 April 2020 May 2020 June 2020 July 2020 August 2020 September 2020 October 2020 November 2020 December 2020 January 2021
Sex/gender—Close split for males/females, but also nice to have Transgender or other if possible.	Male Female Transgender Other
Age range—Spread across age ranges as best as possible—e.g., 3 under the age of 40; 2 between 41 and 55; 2 between 56 and 70; 3 that are 71 and older.	18–24 25–40 41–55 56–70 71–85 >85
Indigenous, or of any other visible minority—Would be nice to interview at least one person if possible.	
Where a person lives—Split between urban and other areas around the city does not have to be equal—may have more from other areas than the City of Calgary.	In Calgary Surrounding communities/rural areas (e.g., Airdrie, Strathmore, etc.)
Complete the telephone survey regarding hospital experience between June 2020 and the end of January 2021.	No need to complete the survey

Patients selected and called by qualified surveyors were invited to participate in the interview study. A structured script was used to guide the call. As patients agreed to talk about their experiences being hospitalized with COVID‐19 and transitioning home to recover, a schedule of selected interview times was provided to them, so they could choose what day and time would work best for them to be interviewed. Study and consent information was also emailed to each of the latter patients. A guide for frequently asked questions was also prepared for the telephone surveyors should they be asked questions about privacy, the interview process and the focus of questions.

### 2.4 Interview process

All interviews were conducted using a secure AHS‐licensed Zoom video platform. Each patient who agreed to an interview was contacted by the qualified interviewer (K. K. B.) to review the study information, consent letter and process, including instructions for the Zoom video. Before commencing the interview, the interviewer and patient discussed the process and the consent to participate, including the approval for having the interview recorded as well as having a note taker present to aid with accurate data gathering and analysis. The comfort level of patients with the note taker in the background was also confirmed, to alleviate the potential impact this may have on patients as they shared their experiences.

A semi‐structured interview guide (attached as Table 3) was used for the 60–90 min interviews. Leading with empathy, questions began with where patients were at with their ‘current status of being at home recovering or recovered’, followed by ‘discharge from hospital’ and ending with their ‘in‐hospital experience’. All interviews were conducted by the same person (K. K. B.) to maintain consistency in approach with questions and content discussion along with observations, as part of in‐depth interviewing. Although each interview was video‐recorded, previous challenges with inaudible recordings were a strong reason for having notes taken during each interview. However, it was also a concern that participants may feel that the interviewer while taking notes was distracted or inattentive to patients as they shared their personal stories.^25^ Therefore, having another person as a note taker allowed the interviewer to give undivided attention to patients while at the same time, all key points and observations were being captured. These key points and observations when compared with the recordings and the transcripts would provide better consistency for the qualitative data analysis, as conducted by both the interviewer and note taker.^26^

**Table 3:** COVID‐19 patient's experience of the semi‐structured interview guide (including prompts)

Focus/major topics to cover	Potential interview questions/focus
Current status while at home	Review of interview purpose, confidentiality, options to answer questions comfortable with and consent confirmation 1. Let's talk about how you are doing now/at this point in time. How are you feeling … physically… emotionally…. mentally? *How well do you feel you are recovering back to your ‘normal self’ (i.e., compared with what you were like before you got COVID‐19)?* *What, if any lingering or new issues are you experiencing at this time (e.g., being short of breath climbing upstairs or doing usual activities; pain; fatigue; mental fog; difficulty walking without help or a device like a cane/walker)?* 2. What, if any, assistance or supports are you using to help you manage your household tasks or other activities, such as returning to work, exercising, childcare, going for groceries, etc.*?* *Any physical assistance/supports including physical therapy or devices to assist you including any mobility supports…?*. *Any other supports to help you emotionally, mentally?* *How did you find these supports? Who was/is involved?* *Probe about role/involvement of family/caregiver in assisting person*. *How well do you feel that your needs are being met or managed?* *What assistance/supports do you feel you are missing or would find helpful? Do you need assistance accessing support/services you need?* 3. What, if any, assistance or support are you using to manage your self‐care activities, such as washing/bathing, dressing, meal preparation; emotional self‐care/meditation etc.? *Physically, emotionally, psychologically* *How did you find these supports for self‐care? Who was/is involved?* *Probe about role/involvement of family/caregiver in assisting person*. *How well do you feel that your self‐care needs are being met or managed?* *What assistance/supports do you feel you are missing or would find helpful? Do you need assistance accessing support/services you need?* 4. What contact and follow‐up care have you had with your family doctor or other healthcare providers since arriving home from the hospital? *Explore what that was like for the patient—if they were contacted, when did that occur and did they appreciate having their family doctor f/u; if they were not contacted…. How did it make you feel?* *Did your family doctor know that you had COVID‐19 and were hospitalized?* *Did your family doctor reach out to you, or did you call your doctor?* *Was a follow‐up appointment with your family doctor prearranged for you before you left the hospital? If so, how did this make you feel?* *If you have not had any follow‐up, what information are you missing? How do you feel about this?*
Discharge from hospital	5. Thinking back to the day you left the hospital to go home—How prepared did you feel to go home from the hospital (i.e., physically, mentally, emotionally and psychologically)? 6. What specific information, help or resources do you remember getting that helped you feel ready, or prepared you for going home? How helpful were these for you? *My discharge checklist?* o *Prevent the spread of coronavirus* o *How to care for a COVID‐19 patient at home* o *Coronavirus disease (COVID‐19): Care instructions* o *Coronavirus disease (COVID‐19): How to manage symptoms)* *Other information given on how to manage problems and progress back to your usual household and self‐care activities while at home?* *Contacts for when you are concerned or have questions or for assistance and support* 7. Looking back, what other information or supports would have been helpful for you to receive before going home from the hospital? *e.g., Who to contact if you needed help or information once you were home; How or where to access supports/services you need* *could also reflect on survey responses as examples: 57% had enough info; (concerns around transportation, costs, out of town; testing positive after isolation; accommodations, isolation)* 8. What suggestions do you have for making your discharge and transition home from the hospital smoother or easier? *Probe using examples from survey responses (medications, isolation, transportation)*
In‐hospital experience	9. Let's go back and talk about your hospital experience—what would you say was one… ‘good’ experience (i.e., went well for you; or something that you appreciated the most?) poor or negative experience for you? (e.g., challenges, upsets, stressors) 10. What, if any supports were offered to you during your hospital stay—i.e., emotional, spiritual, and/or cultural? *If some were offered…. What specifically did you find helpful?* *If none were offered…. What would have been helpful for you? (e.g., SW, psych, spiritual care, Indigenous liaison, navigators, etc.)* 11. How would you suggest the experience of someone in hospital with COVID‐19 and isolated, could be improved? *Probe examples from phone survey results (staff gowning on/off several times; outside food not allowed; no information/news/family contact; not recognize staff/faces personal protective equipment; left alone without any information for long times)* *Offered a device to connect with others*

*Note*: Italics represent potential probes.

### 2.5 Data analysis

The interviews were all transcribed. Thematic analysis was conducted with each transcript,^27^ with inter‐rater reliability checks in place. One analyst used NVivo for coding and theming,^28^ while the second person (K. K. B.) manually coded and themed. Inter‐rater checks were conducted to ensure consistency in codes and evolving themes between the two individuals and approaches applied. Any discrepancies were discussed and addressed by the two raters. Data sufficiency^24^ as well as saturation were determined through two main factors—base number of interviews with consideration of the inclusion criteria, and run‐length of new information analysed to be between 0% and <3%, compared with the majority of other common repeated codes, themes and related information.^29^ Analysed transcript codes and themes were also triangulated with the note‐taker and observation notes, to confirm the accuracy of codes and themes captured and data saturation.^29^, ^30^

### 2.6 Ethics approval

As per protocol for studies classified as quality improvement and based within AHS, the interview process and guide were taken through an expedited ethics review using the ARECCI (A pRoject Ethics Community Consensus Initiative) screening process. The study including the process and interview guide was approved.

## 3 RESULTS

Data saturation was achieved with the 12 completed and thematically analysed interview transcripts. The findings are presented by themes to reflect common experiences among some or most of the 12 patients, with some unique single‐patient experiences. The manner of presentation reflects the aim of the study focusing on patients' experiences during their recovery from COVID‐19 starting with their current state of well‐being and recovery at home and reflecting on their hospital experiences and their readiness or preparation to be discharged and transition from hospital to home.

### 3.1 Profile of patients

Table 4 provides a summary profile of the 14 patients called, as aligned with the inclusion criteria in Table 2 (i.e., hospitalization during different months of the pandemic between March 2020 and February 2021, and by age, sex/gender, ethnicity and where they lived in the Calgary Zone). Two of the fourteen did not complete the interview (i.e., too sensitive or emotional). The 12 interviewed patients were randomly assigned P1–P12 identifiers to maintain their anonymity. Table 4 also contains more details for each patient, P1–P12.

**Table 4:** Profile of patient participants by inclusion criteria

Inclusion criteria	Total number of patients (interviewed and not interviewed)	Individual patient profiles											
		P1	P2	P3	P4	P5	P6	P7	P8	P9	P10	P11	P12
Month													
March 2020	1	**X**											
April	1			**X**									
May	1					**X**							
June	1 Not completed												
July	0												
August	1				**X**								
September	2; 1 Not completed											**X**	
October	1									**X**			
November	3		**X**						**X**		**X**		
December	2						**X**						**X**
January 2021	1							**X**					
Sex/gender													
Male	9; 2 Not completed	**X**		**X**			**X**		**X**		**X**	**X**	**X**
Female	5		**X**		**X**	**X**		**X**		**X**			
Other	0												
Age													
18–24	0												
25–40	2	**X**					**X**						
41–55	2								**X**		**X**		
56–70	6; 1 Not completed			**X**				**X**		**X**		**X**	**X**
71–85	4; 1 Not completed		**X**		**X**	**X**							
>85	0												
Ethnic identification													
White	10; 1 Not complete	**X**	**X**		**X**	**X**		**X**		**X**	**X**	**X**	**X**
Asian	3; 1 Not completed			**X**			**X**						
Black	1								**X**				
Where one lives													
Calgary	11; 1 Not complete	**X**	**X**	**X**	**X**	**X**	**X**		**X**	**X**	**X**		**X**
Surrounding area	3; 1 Not completed							**X**				**X**	

*Note*: Total number of patients called (14), interviewed (12) and not completed (two); and 12 individual patient profiles (P1–P12).

### 3.2 Starting the conversation where patients are at—Their current status recovering at home

Patients' current state of recovery was summarized into six themes—‘Back to Normal Status’, ‘Physical Impact’, ‘Psychological/Emotional Impact’, ‘Personal Life Changes’, ‘Support Needs’ and ‘Follow‐up Contacts’. See Table 5 for the themes, codes and key points regarding the current status of patients recovering at home.

**Table 5:** Themes and codes with brief descriptors

Recovery at home	
**Themes**	**Codes and descriptors**
**Feeling back to normal**	**Degree/extent of feeling/being back to a pre‐COVID normal state—varied across individuals**; when they had COVID over the past year did not factor into their recovery state; some felt back to pre‐COVID ‘normal’ state; recovery related to COVID infection (minor vs. severe), side effects (younger patients) and possibly age; some have medical conditions as well.
**Physical impact**	**Most identified some level of fatigue** (10—generally feeling tired but some feel exhausted; associated with **other physical issues**—shortness of breath; loss of taste/smell; headaches, muscle/back pain, brain fog/memory loss and not being able to sleep. **All trying to get back into normal activities** as much as possible. **Some greater impacts**—difficulty exercising; exacerbated asthma; hair loss, heart and other conditions; some need more one‐on‐one supports or medical care/treatment.
**Mental, psychological and emotional impact**	**Most indicated being fine/okay**—general frustration with COVID. **Some had family/friends impacted**—deaths identified. **Personal issues/concerns**—depression, anxiety, sleep difficulties and PTSD resurgence in one person.
**Spirituality**	**Identified by a few individuals**—prayers
**Personal life changes**	**Realization of need to make changes**—slowing down, prioritizing things differently, changes in eating habits, lower tolerance for foolishness. Most **grateful to be alive**.
**Supports needed**	**Most are not in need of external services or supports**—most have family or friends to assist/support. **Different arrangements**—those living alone have friends or home care supports, meals on wheels, etc.; one person self‐manages and accesses helplines/clinics.
**Follow‐up/contacts**	**Contact with family or other doctors** **Follow‐up varied**—many had calls from family doctors/specialists, some having one call, others many calls; one person did not have a family doctor. Some had specific referrals or follow‐up tests. **Follow‐up calls from Alberta Health Services (AHS)**—few individuals received calls from AHS as a follow‐up; family members connected with AHS for advice or what to watch for.
**Suggestions**	**Standing protocols for follow‐up with patients released from the hospital** Either AHS or a family doctor is scheduled to call patients. Check on the status and needs of patients.

*Note*: Current status of patients recovering at home.

All individuals claimed that they were recovering but each differed in terms of the degree or extent to which they felt or believed their health or condition was back to the pre‐COVID‐19 ‘normal’ state. For example, two individuals claimed to be back to ‘normal’. A few felt that their recovery was related to whether their COVID‐19 infection was ‘more minor versus more severe’ (P4), while others thought that their recovery was taking longer because of the impact of other medical conditions like heart failure, injuries (i.e., fractured shoulder), or side effects of COVID‐19. Two others attributed their slow recovery to their age (‘not quite sure whether that I'm getting older, or is it COVID i.e.,—is holding me back’—P3). The timing of their hospitalization, whether early 2020 or later did not factor in with their perceptions regarding their recovery progress or current state. More specifically, those individuals who did not feel their recovery was back to ‘normal’ commented on the lingering impact and issues of COVID‐19 on their physical as well as mental health, including their psychological, emotional, spiritual and social well‐being.

Physically, most individuals (*n* = 10) identified some level of ‘fatigue’ (‘…the fatigue, that comes and goes, there is no rhyme or reason for it. One second I'm feeling fine then next minute I have to sit down’—P12). Some described feeling ‘exhausted’ (P2), mostly associated with ‘shortness of breath’ (P3, P5, P6, P8, P12) having a greater impact on them. Mentally (i.e., psychologically and emotionally), most indicated they were fine or ‘okay’ (P4) or ‘didn't feel seriously ill with COVID’ (P11). Five individuals had family members who also had COVID‐19, and while most were grateful they were okay or recovering, one person experienced the loss of a spouse due to COVID‐19 complicated by other medical conditions. One other person lost two acquaintances to COVID‐19, linked to a church service where COVID‐19 spread and infected more than half of the congregation members. Individuals noted a variety of specific personal issues/concerns, including depression, ‘lack of self‐motivation to get up’ (P10), anxiety attacks, sleep difficulties, difficulty exercising, hair loss, headaches and brain fog, among other symptoms. ‘The brain fog is consistent; my ability to keep track of things has gone out the window… I have to literally write everything down to make sure I don't make mistakes’ (P12).

Half of the patients also noted that their experience with COVID‐19 made them realize that it was time to make some changes in their lives and practices, which included ‘slowing down’ (P12), prioritizing things differently (i.e., pre‐COVID‐19 issues were no longer concerns) and changing the eating habits. Most individuals claimed that they ‘feel lucky, grateful’ (P2) to be alive.

Regarding the need for extra supports, most (10 of 12) individuals indicated that they were not really in need of extra external services or supports, but also knew of or had access to some support. These individuals had family at home to assist or had friends or other supports (e.g., Home Care services) already arranged to assist them. For example, those with family members at home had meals prepared, shopping done or delivered and other household tasks attended to. One person was a caregiver to a spouse and had to manage both of them (‘I'm a caregiver for my husband, so I have to stay okay’—P5). Others who lived alone had friends who helped (i.e., grocery shopping, delivering prepared food, getting medications, etc.), or had Home Care services previously in place and continuing, or meals on wheels arranged. Individuals with heart or other medical conditions had one‐on‐one supports or medical care/treatment as prearranged. For seven individuals who had family doctors, their experiences with follow‐up regarding COVID‐19 and/or other conditions were different. In total, 11 individuals had received one or more calls from their family doctors or other specialists after getting home from the hospital. Four of these individuals also mentioned receiving follow‐up calls from AHS transition services staff. A few patients had spouses or family who knew to call the AHS Help Line for advice or what to watch for.

### 3.3 Reflecting back on the patients' discharge from hospital—Their readiness and preparation

Patients' experiences with their discharge and preparation to go home following hospitalization were summarized under five themes—‘Discharge Process’, ‘Preparation/Feeling Prepared to go Home’, ‘Information/Resources/Supports’ and ‘Improvement Suggestions’ (see Table 6 for the themes, codes and key points).

**Table 6:** Themes and codes with brief descriptors

Discharge from hospital	
**Themes**	**Codes and descriptors**
**Discharge process**	**Notice to patients regarding discharge**—day of or day before. **Process**—confusing for some; criteria for discharge; waiting for sign‐off; contacting family. **Transportation arrangements**—family; one needing taxi/other. **Emotional experience** for some.
**Preparation/feeling prepared to go home from the hospital**	**Varied preparation for discharge and going home**. **Mixed feelings of staff knowledge regarding discharge**—most okay; some feared early release and relapse (three readmitted). **Different/individual medications/oxygen arrangements**.
**Information, supports and resources**	**Inconsistencies in information patients received, how and when**—related to hospital and staff; what was available to give patients. **Eight patients received discharge information/packages**—some opened them, and found the information useful. **Other considerations**—varied staff experiences but overall good job.
**Other information or supports needed**	**Those with discharge information** did n*o*t need anything else. **Some wanted better pre and during discharge** information and related discussion/explanation for postdischarge follow‐up. **Those not getting any information** did not know what they missed.
**Suggestions for making discharge or transition home smoother**	**Better verbal communication** by staff before/during discharge recondition, contact information and follow‐up directions. **Retest patients** to ensure they test negative before going home. Staff have the same **consistent messages during redischarge/follow‐up**. **Postdischarge protocol needed for checks on patients** by doctors and/or AHS.

*Note*: Patient experience with their discharge from hospital and preparation to go home.

Abbreviation: AHS, Alberta Health Services.

The actual notice or communication from staff to patients regarding their discharge from the hospital came either the day before or the day of discharge. Information provided was inconsistent across patients. For some patients, this process was confusing as hospital staff did not seem to be well informed about the process (‘miscommunication regarding discharge paperwork or follow up’—P10*)* and/or had to wait for the doctor to sign off. This meant delays or changing arrangements with family or friends for transportation home. Most patients had family members or friends with whom they made arrangements for transportation home, but one person had no way to get home and thus waited a long time for a taxi. This was a highly emotional experience.

…there was a service I could use within Alberta Health Services to get myself home, the problem was nobody at the hospital knew about it…The people at EMS [Emergency Medical Services] were pushing back about allowing me to use it. …that was a major anxiety provoking moment, to be stuck in a hospital with no way home, and not knowing if you*'*re going to get home…. I was so anxious I was shaking. Took an hour and a half for the taxi to show up, I was literally crying when I got in the back of the taxi, thanking the driver for coming to pick me up. That should never have happened. (P12)

Four individuals said they were well prepared to be discharged to go home. The others were told and understood that they had to meet specific criteria to be granted a discharge, including walking/mobility, breathing checks and medications working as intended. Most felt that staff were knowledgeable about these latter aspects and trusted them. However, three individuals felt they were discharged too early (i.e., felt unwell, exhausted and struggled to breathe). These three experienced relapses and were readmitted within 24 h after their discharge, two due to low oxygen saturation and another because of anxiety/panic attacks affecting heart rate and breathing.

Patients had different experiences when it came to medications or oxygen they needed for their recovery at home. For example, one individual had a prescription faxed to a pharmacy and had to find a way to pick this up. Another person was provided enough medication to take with him and use while at home. One other person had to make an unscheduled trip back to the hospital to sign documents regarding arrangements for the oxygen to be retrieved from his home after it was used up. This latter patient indicated that the staff person checked and told him he was able to leave, but unfortunately, that was not the case:

…nurses that came to tell me, ‘you know you're done, you're free to go … discharge papers have been filed’. I specifically asked them ‘shouldn't I wait for the lady who does the oxygen tanks to come and see me and take my information?’ They went away checked …. and said ‘no you're fine that's all taken care of’. I got my ride to come to the hospital and pick me up, got into the car and we headed off down the road, and before we'd gotten more than about halfway home the phone call comes in and says we need you to come back to the hospital; you need to give your information to the oxygen lady. (P10)

There were also inconsistencies regarding what information different patients were given, how it was given and when and by whom. Different hospitals had different approaches to discharge, and patients had mixed feelings about staff being prepared or knowledgeable about discharge practices. The timing of when patients were hospitalized may not have made a difference in what information patients were given or how. During the early pandemic, some patients got handwritten information while others got packages. Some individuals hospitalized later in the pandemic did not receive or did not recall receiving discharge packages or much of any kind of information. Of the eight patients who said they had received some discharge information or packages, half noted the different types of information, including contact information for doctors or others, information on COVID‐19 and protection and self‐management advice. They found the information useful—‘when I first got it [the discharge package] …I found it answered my questions…I always check the information packet first before bothering to call 811’ (P10). The other half recalled getting a package but never opened it; some had kept the package in case they needed it. Those that did not receive any information felt it was because there was not much information available about COVID‐19 and what to do, as well as a lack of experienced staff. Patients generally commented on a good job of staff managing things during COVID‐19 and providing what information was known at that point in time. Individuals who had received discharge information felt there was nothing more they needed. Others felt that they needed better pre and during‐discharge information and better discussion or explanation regarding postdischarge follow‐up—that is rather than just being handed a package, staff should explain what it contains and what patients could do or what they could expect as a follow‐up. Some were uncertain of what information they missed as they had not received any before their departure from the hospital.

Generally, most patients did not want to complain and felt the nurses and doctors did all they could, given what they knew and could do at specific times during the pandemic. Others had a few suggestions based on their specific experiences during discharge and with their home follow‐up. These patients suggested that verbal communication by staff (i.e., nurses and/or doctors) before and during discharge could be better, whether the patient received a discharge package or not. Specifically, patients felt they needed to be informed as to their condition to go home, and then given clear follow‐up contact and other information including what the written materials contained—that is, ‘there be better discharge education’ (P12). A few other specific suggestions were made, including that: patients be retested to ensure they are negative before going home to family; all staff repeating the same consistent messages regarding discharge and follow‐up (i.e., ‘otherwise confusing as to who to believe’—P9); and AHS incorporate a postdischarge protocol that includes AHS staff or doctors following up with patients to check on their status or needs (‘…some follow up would have been good’—P3*)*.

### 3.4 Patients' hospital experiences—Care influencing recovery

Patients' experiences with their in‐hospital care and supports were captured under four themes—‘Intensive Care’, ‘COVID‐19 and General Care’, ‘In‐hospital Supports’ and ‘Improvement Suggestions’. Also relevant to patients' in‐hospital experience, two additional themes were captured—‘Prehospital Care’ and ‘Emergency Department (ED) Care’ (see Table 7 for the themes, codes and related experience points captured).

**Table 7:** Themes and codes with brief descriptors

In‐hospital experience	
**Themes**	**Codes and descriptors**
**Prehospital experience**	**Contact with AHS**—advice re: symptoms was usually go to hospital; some patients given misleading information—passed off for flu. **Prehospital**—Positive for most patients accessing/using **EMS**. **Questionable advice** sometimes given to patients.
**Emergency department experience**	**Different experiences for each person**—worst part of most patients' hospital experience (not related to staff but to processes and long waits); some patients feared their medical conditions would be overlooked. All indicated **need to have someone check on patients regularly**.
**Intensive care experience**	**Those in ICU felt well taken care of**—good interaction; family allowed in; staff responsive to patients; options for treatments provided and explained; information given; questions answered.
**COVID general care**	**Patients viewed experiences as good**—most felt they got good care; staff competent, knowledgeable and well trained. Most patients **did not see doctors but nurses** were personable, helpful and addressed needs. **Preference for private rooms**. **PPE protocols made patients feel safe**.
**Poor/negative experience**	**No major complaints but some negative experiences**. **Isolation**—no family allowed; feeling isolated even with staff around; no TV or access to iPads for some—had to have family/friends drop off iPads. **Food**—mixed views **Not having a private room**—sharing generally and one with person of opposite sex. **Nurses refusing to don PPE** to bring in water, Tylenol, food, blankets—left items by the door for patient to retrieve; call bells not answered when needed.
**In‐hospital supports**	**Minimal/no supports**—because of COVID, most supports like therapies, etc. were not available/accessible. **iPads limited in number**—patients needed to have someone deliver their iPads, etc. Some rooms had TV, most did not. Personal care items provided to patients, as needed.
**Suggestions for improving patient hospitalization experience**	Address **food** issues. **Keep family members with COVID together, if possible**. Staff/doctors need to **keep patients/families informed**; doctors should make point of seeing patients. Improve the **professionalism of some staff**—reissues with PPE. Attend to **patients' medical conditions** as well as COVID. **Retest patients before or at the time of discharge**.

*Note*: Patient experience with hospitalization.

Abbreviations: AHS, Alberta Health Services; EMS, Emergency Medical Services; ICU, intensive care unit; PPE, personal protective equipment.

Many patients shared their prehospital experience including calling AHS Help Line for information and either being driven or taken by ambulance to the ED at one of several hospitals. In the ED, each individual's experience was different, but most indicated that this was the worst part of their hospital experience overall. They commented on the ED being a super busy place, lots of noise, ‘lack of true privacy’ (P5), feeling isolated (‘very lonely experience without family or anyone for support’—P7), and ‘long waits to talk with doctors’ (P8) as well as getting assessments, tests/test results, and other information before being admitted or discharged. All patients indicated the need to have someone (e.g., HealthCare Aid) check on every patient every so often to determine status, help with needs or just to ask ‘How are you doing?’ (P6).

Those who went to the intensive care unit felt well taken care of. Family members were allowed to come in. Staff were very responsive to patient and/or family call bells and needs—information was provided, questions answered and encouragement given to patients to get up when they were feeling better and walk around their room. Half of the patients interviewed viewed their experiences on the general COVID‐19 units as generally good—‘I'm not a big fan of hospitals…it was such a great experience… I mean I still remember the one nurse, … he like went through a whole pile of pillows to find me the most comfiest pillow’ (P1). Everyone commented on how bad it was not having family allowed due to visitation restrictions, although they understood the reasons for the policy. Also because of the restrictions in place, there were very few or no supports including physio or other therapies available or accessible.

Aside from the food, most individuals interviewed did not have any suggestions for improvements in in‐hospital care. Their experiences were generally positive and any minor issues were because the staff were overstressed, but doing their best under the circumstances—‘wonderful care given’ (P2, P4). A few felt differently, particularly with how staff communicated with patients: ‘…the mechanical nature of care was the problem, it lacks warmth; it lacks compassion. Now some of that could have been enhanced by personal protective equipment (PPE) and everything like that. I get all that, but overall, when I stripped away the PPE…I was a number and knew it’ (P12). This included not having doctors see patients and answer questions that nurses could not. Some interviewed commented on the professionalism of some staff asking patients to get up to get their own items of care (i.e., Tylenol, water chips) from the floor at the door or ‘have blankets thrown at them from doorways’ (P9). These patients were made to feel guilty for asking for assistance—‘I felt bad asking for something [be] cause they had to garb up every time they came into our room’ (P5).

## 4 DISCUSSION

Through this qualitative study involving 12 patients who had COVID‐19, we set out to understand their experiences recovering at home and reflecting back on how their hospital care and discharge prepared them to transition home. This reversal in the interview approach allowed us to hear about their present situation, as they told us what their recovery circumstances were like, and how the actual preparation, supports, resources and information they received as part of their discharge process from the hospital, as well as hospital experience, helped shape their journey and experiences to recovery.

Our findings, like those in the literature, supported the fact that recovery from COVID‐19 to pre‐COVID‐19 ‘normal’ state varied considerably for different individuals, including any lingering physical, psychological, mental and emotional symptoms.^1^, ^2^, ^3^, ^4^ Individual recovery was also associated with their general health, existing medical conditions and age,^20^ as much as the severity of the COVID‐19 infection.^2^, ^3^, ^4^ For some patients, the long‐term or long‐haul implications of COVID‐19 impacted their quality of life and their capacity to resume pre‐COVID‐19 ‘normal’ activities, including work, school, hobbies and physical activities.^2^, ^3^, ^4^ As varied as their circumstances and recovery from COVID‐19 were, patients dealt with and managed in different ways, most dependent on supports from their families, caregivers or friends, and either prearranged Home Care or medical care follow‐up, or seeking advice when they needed it (e.g., through AHS Help Line). Patients in this study suggested that there be follow‐up calls from either family doctors notified of their patients' COVID‐19 status or from AHS staff (e.g., discharging hospital transition coordinators). This aligns with other studies clearly indicating the need for regular monitoring of discharged patients to assess clinical status as well as the need for medical or rehabilitation care.^12^, ^13^, ^14^, ^15^ Approaches for follow‐up with those patients having other medical conditions were different. They had virtual care and monitoring by their family doctors or specialty healthcare teams including ongoing assessment of their COVID‐19 status and additional supports or treatment as needed. This type of monitoring also reduced the likelihood of them being readmitted to the hospital.^17^, ^18^, ^19^

The discharge from hospital preparation protocols and processes,^12^ including information provided, how, when and by whom, seemed to be key in establishing patient and/or family/caregiver or other capacities for managing recovery at home.^12^, ^13^, ^14^, ^15^ Yet, for many patients, this was a less than positive and rather confusing experience.^9^ Much of this confusion could be attributed to the inconsistencies in discharge assessments and notifications, information for arranging transportation or follow‐up care, as well as inconsistencies in the way these various aspects of discharge were communicated to patients and/or their significant others,^10^, ^11^ and particularly to those with other medical conditions.^20^

Patients in this study as in others made some clear suggestions for improvement around the discharge assessment, preparation and follow‐up process. Most patients targeted the need for better verbal communication and even patient education by staff regardless of any written information or packages provided.^12^, ^13^, ^14^, ^15^ Informing patients about their time of discharge and making transportation arrangements were crucial to preventing confusion or delay.^10^ Also important were the community or other contacts and information for recovery care or rehabilitation, which some or many patients would need to know how to access. AHS did have discharge information sheets, packages and post‐COVID‐19 Help Lines in place early on in the pandemic, which should or could have been shared and explained by staff with every patient at the time of their discharge from hospital to home. A support programme to facilitate discharge home was developed for implementation across AHS through the provincial *Post‐COVID Rehabilitation Strategy Framework*.^31^ This strategy includes patient screening, care pathways and follow‐up through multidisciplinary clinics or virtual programmes much like those suggested in the literature.^16^, ^17^, ^18^

**Figure 1:**
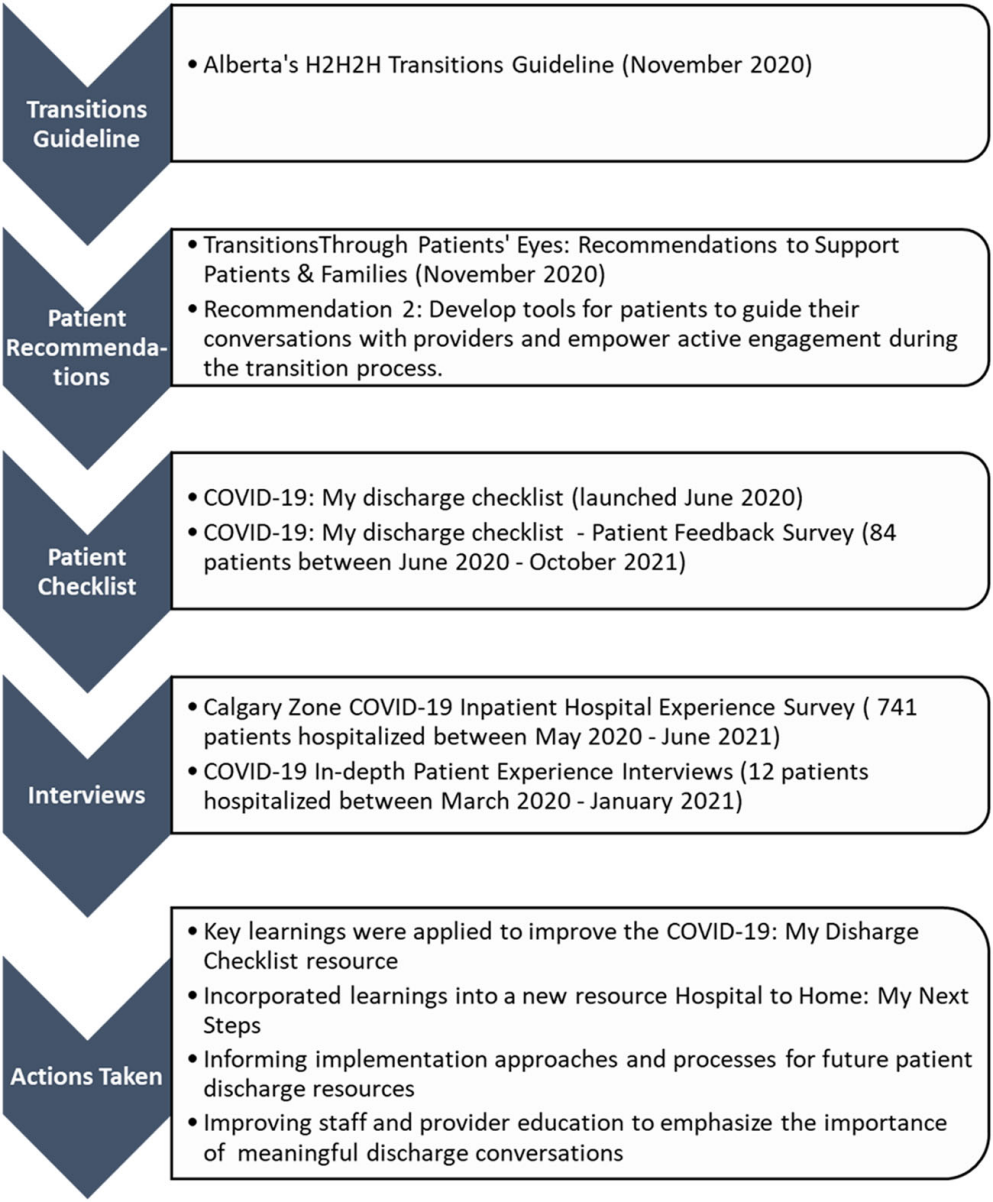
Progression of work related to patient transitions resources: before, during and following COVID‐19 patient interviews.

Our work to support patients to be better informed and prepared for their transition home from the hospital is grounded in the development and implementation of the *My Next Steps: Getting ready to leave the hospital*.^32^ A team co‐led by patient advisors developed *Transitions Trough Patients' Eyes: Recommendations to Support Patients & Families*.^33^ In this latter report, the patients recommended the development of tools for patients to guide their conversations with providers and empower active engagement during the transition process. This guided the team to quickly respond by developing a self‐management resource for patients hospitalized for COVID‐19. *The COVID‐19: My Discharge Checklist* ^34^ patient transitions resource developed with patients for patients was created because visiting restrictions in hospitals prevented patients from having family or essential caregivers present and participating with them in discharge planning conversations. As we learned through the interviews, patients were not always well prepared with the right information to go home; were discharged quickly as cases and hospitalizations rose rapidly; did not feel confident to manage safely at home; did not have clear paths for who to contact regarding concerns or increased symptoms and sometimes needed to return to the ED or be readmitted.^20^ See Figure 1 for a summary of the work leading up to, as well as including and following the COVID‐19 patient experiences interviews.

Much of the in‐hospital COVID‐19 care was viewed by patients as being what it was because of the pandemic and the protocol restrictions in place, but they generally had positive things to say about their experiences,^7^ including those who had to stay in Intensive Care.^8^ The resulting restrictions prevented family visitation^9^ or the provision of therapies and supports, both of which patients relied on for mental health and physical well‐being. Adding to the isolation was the fact that staff also did not spend much time talking with or checking on patients. Patients suggested that staff do more periodic checks on patients to monitor their status and check on their needs. Patients should not have to feel guilty asking for items they needed as part of their care, or having staff ‘garb up’ to see them.

Although more extensive qualitative studies like this one are needed to capture the recovery at home journeys of patients with COVID‐19, including their hospital discharge and transition preparation, the findings from this study have provided some insights supported by and contributing to the literature. Although the perceived limitation of this study, as inherent with many qualitative studies, includes the number of interviews conducted, we were able to demonstrate data sufficiency^24^ and data saturation through our iterative thematic analysis of transcripts.^29^, ^30^ Other noted limitations included that only English‐speaking patients hospitalized between March 2020 and February 2021 were targeted, with some additional consideration for sex, age and/or where the persons lived. Also, patients called could decline or accept the invitation; many patients declined and therefore, patients accepting the invitation were considered to be self‐selected.

## 5 CONCLUSION

Findings from this study not only informed us of the recovery journey of patients with COVID‐19 while at home but also provided a rich understanding of the needs, information gaps and what matters most to patients when they are preparing to safely and successfully transition from hospital to home to recover. Key learnings from the study were applied to improve the *COVID‐19: My Discharge Checklist* ^34^ patient resource, and develop a general patient discharge and transitions resource—*My Next Steps: Getting ready to leave the hospital*.^32^ Patient experiences from the interviews also informed the need for training and education of hospital staff and care providers regarding more consistent and meaningful discharge conversations with patients. This latter part of *Alberta Health Services Post‐COVID Rehabilitation Strategy Framework* ^31^ guides patient as well as multidisciplinary team experiences regarding post‐COVID‐19 screening, monitoring and recovery. Future work is needed to explore how the proposed changes in resources, communication and other initiatives improve patient experiences, self‐management and overall outcomes.

## CONFLICT OF INTEREST

The authors declare no conflict of interest.

## Data Availability

Raw interview data for this study is not available to share as per Alberta Health Services Privacy and Health Information Acts, protecting patient personal and experience data. Cleaned data can be made available upon request.
